# Static-progressive bracing is associated with improved range of motion in patients with post-traumatic and post-operative elbow stiffness at three months

**DOI:** 10.1177/17585732251389182

**Published:** 2025-10-22

**Authors:** Mark F Siemensma, Eline M van Es, Anna E van der Windt, Joost W Colaris, Denise Eygendaal

**Affiliations:** 1 Department of Orthopaedics and Sports Medicine, Erasmus MC, University Medical Center Rotterdam, Rotterdam, The Netherlands

**Keywords:** elbow stiffness, post-traumatic, non-surgical treatment, splinting, static-progressive bracing, turnbuckle brace

## Abstract

**Background:**

Elbow stiffness after trauma or surgery remains challenging to treat, with limited consensus on optimal approaches. Treatment efficiency could be improved by identifying patients likely to benefit from nonsurgical treatment. Therefore, this cohort study assessed short-term changes in elbow ROM and patient-reported outcome measure (PROM) scores during static-progressive bracing in patients with post-traumatic or post-operative elbow stiffness.

**Methods:**

We included patients with symptomatic limited elbow flexion and/or extension, indicated for brace therapy. Elbow motion (flexion, extension, total motion arc), PROM-scores, and treatment satisfaction were collected at baseline and at three months. Differences were analyzed using paired *t*-tests. Possible predictors for poor treatment outcome (≤10° improvement at three months) were identified using univariate regression.

**Results:**

Twenty-nine patients were included. Statistically significant improvements were observed for flexion, extension deficits, total motion arc, and PROM-scores (OES and Quick-DASH). However, both PROM-scores did not surpass the minimal clinically important difference. Overall treatment satisfaction was 56%. Age emerged as a possible predictor for poor treatment outcomes in extension deficits. No brace-related complications were observed.

**Discussion:**

While increasing age may reduce efficacy in patients with extension deficits, static-progressive bracing appeared safe and was associated with early improvement in ROM in patients with post-traumatic or post-operative elbow stiffness and should be considered in all patients.

## Introduction

Elbow stiffness after trauma or surgery remains challenging for physicians to treat. Elbow stiffness is defined as a loss of extension greater than 30° and a flexion of less than 120°.^1^ Rotational impairments may also be present, albeit outside the scope of this article. If left untreated or if treated incorrectly, elbow stiffness can lead to severe limitations in elbow function, which can have a substantial impact on a patient's daily life.^2–4^ Current treatment options consist of physiotherapy, bracing, surgery, or a combination of modalities. However, current literature is still indecisive regarding the most optimal treatment strategy. To prevent patients from unnecessary discomfort and to optimize treatment time, it would be ideal for clinicians to identify which patients will benefit from non-surgical treatment and which patients will not, and thus requiring surgical contracture release.

Physiotherapy is considered the first treatment of choice for the prevention and treatment of post-traumatic elbow stiffness. It can be effective in maintaining range of motion (ROM) after trauma, and improving ROM and reducing pain after the onset of stiffness.^5^ The best results are achieved if treatment is started within six months after the onset of stiffness.^6,7^ Bracing can be added as an additional therapy for elbow stiffness resistant to physiotherapy alone. Although older studies have shown good results with bracing, with the surgical advancements towards arthroscopy in recent years, bracing has receded more towards the background and is sometimes even overlooked as a suitable therapy.^8–10^ However, leaving out brace therapy as a non-surgical treatment would be a major missed opportunity in the treatment strategy for this already challenging condition.

Braces use the principle of applying continuous tensile forces in the direction of the motion deficit over longer time periods. This continuous load allows for elongation of the contracted soft tissues over time. Two types of bracing are mostly used: static-progressive braces (SPB) and dynamic braces. Static-progressive braces use an initial high force to provide stretch in the direction requiring improvement. This force decreases over time due to the elongation of the contracted soft tissues. This requires the SPB to be tensioned every 5–10 min over the course of each session, with multiple sessions per day. Dynamic braces, on the other hand, use a spring-like mechanism to constantly exert lesser force than SPB's in the direction requiring improvement. Current treatment methodologies in brace therapy vary widely in terms of bracing protocols, total treatment duration and in the use of additional rehabilitation exercises.^8–14^ A systematic review previously showed no preference for one brace technique over the other.^15^ The most recent randomized controlled trial (RCT) on braces for flexion and extension showed good and comparable results for SPB with a bracing protocol of three times per day for 30 min and dynamic braces worn for six to eight hours continuously.^13^ Dynamic braces, however, are considered to be more demanding for patients, more expensive and less comfortable than SPBs.^4,13,14,16^ Moreover, longer consecutive time periods of stretch in a single direction are not suitable for all contracture types. In flexion contractures, prolonged stretch in the direction of flexion may evoke ulnar nerve problems, especially for patients with long-standing flexion contractures with a flexion arc less than 90 degrees.^4^

If conservative therapy fails, elbow ROM can be improved surgically by means of open or arthroscopic arthrolysis. Both surgical techniques have become well-established, with comparable results for either technique. While arthroscopic arthrolysis has a lower complication and revision rate, the indications for the technique are narrower, and the technique is considered to be more technically demanding.^4,17–21^ The decision to convert to surgery if insufficient improvement is seen with non-surgical therapy is generally made after three months of treatment with brace therapy. It is, therefore, crucial to investigate whether brace therapy can offer clinically relevant improvements within such a short period. Moreover, possible factors that attribute to treatment-resistant stiffness are still poorly understood. Therefore, factors such as prolonged immobilization and multiple previous surgeries may negatively influence response to treatment.^22,23^ Despite surgery being effective for restoring elbow motion, recurrent stiffness following arthrolysis still is a known and common issue, providing another point in treatment where brace therapy may be beneficial.^24–26^

While improvements in range of motion (ROM) and other clinical metrics during treatment are crucial, these metrics alone do not fully capture the patient's perspective and quality of life (QoL).^27^ The integration of patient-reported outcome measures (PROMs), such as pain, treatment satisfaction, and function scores, provides valuable insights into the patient's perspective on the functional integration of their elbow into daily life and their QoL during and after treatment.^28^ This underlines the necessity of integrating these metrics in treatment evaluations to gain a broader understanding of treatment effectiveness. However, there is currently a scarcity of literature reporting the effectiveness of static-progressive elbow braces on PROMs.

Therefore, the aim of this study is to evaluate the short-term outcomes of our current bracing protocol in patients with post-traumatic or post-operative elbow stiffness. Outcomes will be determined in terms of the increase in elbow ROM and improvement in PROMs.

## Materials and methods

### Study design and population

This study was designed as a cohort study in which the clinical data from routine outpatient clinic visits was entered prospectively into the electronic patient data system (EPDS). We included all consecutive patients in our study with symptomatic limited elbow flexion and/or extension due to post-traumatic stiffness or recurrent stiffness after surgical contracture release, and divided these patients into two groups. The post-traumatic group consisted of patients without any increase in ROM during three months prior to treatment. This plateau criterion was intended to reduce the likelihood that subsequent ROM changes were due to natural improvement. The post-surgical stiffness group consisted of patients who did not improve substantially with post-operative physiotherapy, and patients who showed >15° decline in ROM, compared to the ROM achieved during surgery at their first follow-up visit six to eight weeks after surgery. All patients were included at a highly specialized upper limb unit of a large Western European University Hospital from the 1^st^ of September 2022, with a minimum follow-up of three months. Both adults and children were included. Prior to their first outpatient clinic visit, all patients received digital routine baseline PROM questionnaires. Informed consent was obtained from all subjects involved in the study. Both clinical and PROM-data were extracted retrospectively from the EPDS at baseline and at three months. The study was conducted in accordance with the Declaration of Helsinki and approved by the medical ethics committee of the Erasmus University Medical Center, Rotterdam (approval ID: NL-009382).

**Figure 1.**
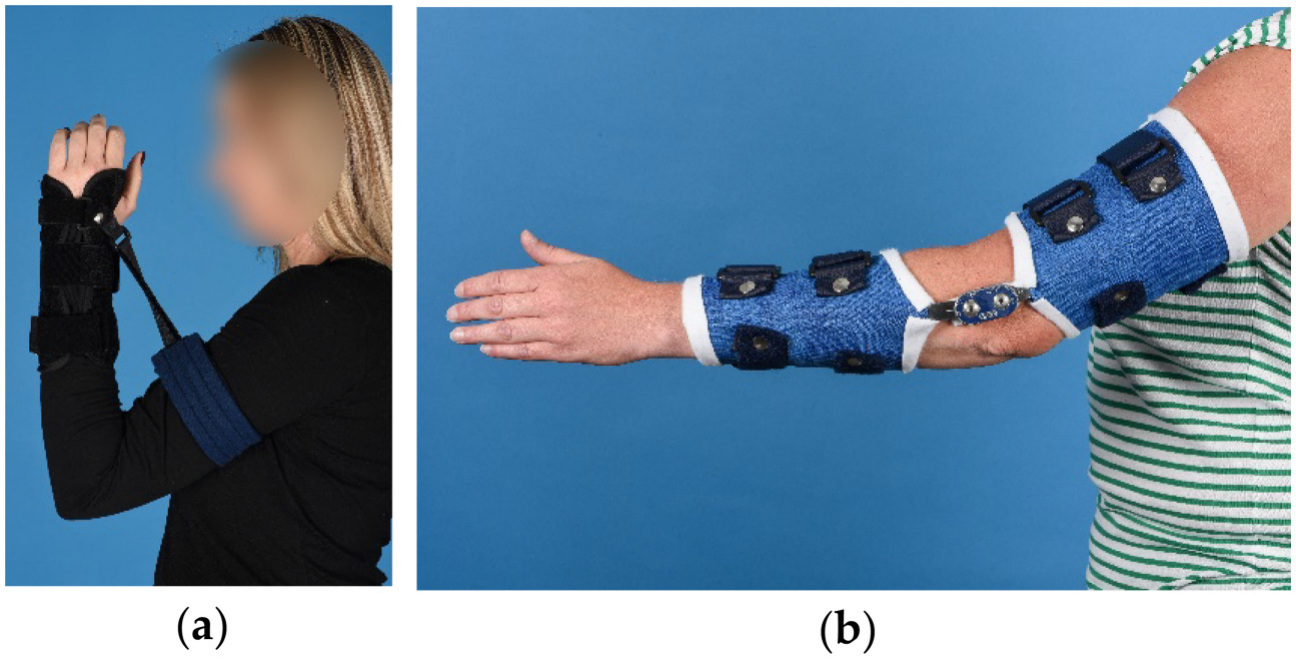
Static-progressive brace types used for treatment of elbow contractures. (a) Brace with strap and loop design, used for treating flexion deficits; (b) Turnbuckle brace, used for treating extension deficits.

Based on literature, own experience, material availability and expertise of our brace technicians, two different brace types were used in our treatment protocol.^4^ Patients with a flexion deficit wore a brace with a strap and loop design for three times 30 min daily (Figure 1(a)). Patients with an extension deficit wore a turnbuckle brace for eight hours consecutively, preferably during the night (Figure 1(b)). Patients with a deficit in both directions wore their flexion brace during the day and their extension brace during the night. Brace-wear adherence (time-in-brace) fidelity to the brace wearing protocol and formal metrics of brace tolerability were not systematically captured. Concomitant physiotherapy was permitted as part of routine care. The exact exercises and therapy frequency were left at the discretion of the treating team and were not systematically recorded.

### Primary objectives

For our primary objective, elbow flexion, extension and total ROM-arc were measured by the treating orthopedic surgeon (DE, JC, AvW) using a hand-held goniometer, with angles read to the nearest 5°. Scores at three months were compared to baseline. Baseline was defined as the date at which brace therapy was started.

### Secondary objectives

Secondary objectives were the change in PROM-scores for the Oxford Elbow score (OES), Quick Disabilities of the Arm, Shoulder, and Hand (Quick-DASH), and Patient-Specific Functional Scale (PSFS) questionnaires. Furthermore, patient satisfaction scores and pain scores at rest and after activity were extracted from the EPDS. Scores at three months were compared to baseline. All PROM-scores were obtained following routine care in our hospital. For patient satisfaction, patients were asked how satisfied they were with their treatment on a six-point Likert scale ranging from utmost unsatisfied to utmost satisfied and subsequently graded their elbow (elbow grade score) on a scale between 0 and 10, with 0 being the lowest satisfactory result and 10 being the highest satisfactory result. Pain was measured at rest and after activity using a visual analog scale (VAS), with 0 being no pain and 100 being the highest imaginable pain. In addition, brace-related complications were defined as adverse events attributable to the device requiring medical intervention (e.g., skin trauma, neuropathy) or as complete discontinuation of treatment and were subsequently registered. Furthermore, factors such as age, sex, time to intervention (period from trauma or previous surgery to start bracing), pain scores at rest and after activity, previous immobilization (cast or external fixator), and multiple surgeries were analyzed as possible predictors for poor treatment outcome. Poor treatment outcome was defined as ≤10° improvement in flexion or extension deficit at three months.

### Statistical analyses

Continuous data were analyzed in terms of means and standard deviations for normally distributed data. A *p*-value of <0.05 was considered statistically significant. Differences in flexion, extension deficit, total ROM-arc, OES, Quick-DASH, PSFS, patient satisfaction, and pain at rest and after activity at three months compared to baseline were evaluated using paired *t*-tests. Possible predictor variables for poor treatment outcome at three months were identified by means of univariate regression using generalized linear models (GLMs).

## Results

### Baseline data

In total, 29 patients were included in our study, of which 10 males, and 19 females. Average age was 32 years (range 7–67 years). Seven patients were treated for their flexion deficit, nine patients were treated for their extension deficit, and 13 patients were treated for a deficit in both directions. Eighteen patients were treated with brace therapy in the post-traumatic group, and 11 patients were treated with brace therapy in the post-surgical group (Table 1). The overall mean time between initial trauma or surgery and the start of brace therapy was 21.4 months (range 3 months–16.1 years).

**Table 1.** Overall patient demographic data.

Characteristics	No. or mean	% or range
Patients		
Total	29	100
Adult	20	69
Pediatric	9	31
*Sex*		
Male	10	34
Female	19	66
*Age, years*		
Adult	47	22–67
Pediatric	11	7–16
*Brace type*		
Flexion	7	24
Extension	9	31
Both	13	45
*Stiffness type*		
Post-traumatic	18	62
Post-surgical	11	
OA	7	24
AA	4	14

OA: open arthrolysis, AA: arthroscopic arthrolysis.

### Subsequent treatment

In the post-traumatic treatment group, four out of 18 patients underwent a surgical procedure in addition to brace therapy. This was either due to insufficient improvement with brace therapy alone (two patients) or due to insufficient improvement combined with either hardware complaints (one patient) or the development of periarticular ossifications (PAO) (one patient). One of two patients who showed insufficient improvement with brace therapy alone was only able to wear their brace at night for 2–3 h due to too much pain. This was categorized as a tolerability issue, rather than a complication, since it did not involve complete discontinuation of treatment. The patient having hardware complaints required open arthrolysis together with hardware removal. Lastly, the patient who developed PAO also had a nonunion after 8.5 months following an olecranon fracture and was treated with open reduction and internal fixation (ORIF) together with open arthrolysis and PAO removal. To ensure that the outcomes reflected brace therapy alone and were not influenced by additional surgical procedures, these four patients were therefore excluded from further analysis.

In the post-surgical group, brace therapy was started earlier (between two and three weeks) after contracture release in three patients. These patients all had nerve complaints related to the trauma prior to surgery (one ulnar nerve hypoesthesia, one radial nerve dysfunction, and one delayed-onset ulnar neuropathy [DOUN]). Brace therapy was started directly post-operatively in one patient after surgical contracture release, together with the removal of their radial head prosthesis. Besides one patient, all patients received at least three months of brace therapy after surgery. The patient who did not receive three months of brace therapy after surgery discontinued brace therapy at six weeks due to osseous impingement following PAO. Both the PAO and the residual stiffness were treated without further interventions since the patient did not want additional surgery.

### Primary outcomes

Statistically significant improvement was seen in elbow flexion, extension deficit and total ROM-arc at three months. Mean elbow flexion improved from 109° to a mean of 121° (*p* = 0.001). Mean extension deficit decreased from 27° to 18° (*p* = 0.002), and mean total ROM-arc improved from 83° to 102° (*p* < 0.001). An overview of our primary outcomes is shown in Table 2.

**Table 2.** Differences in flexion, extension deficit and total ROM-arc at baseline and at three months.

ROM type	Mean at baseline	95% CI	Mean at 3 months	95% CI	*p*-value
Flexion	109°	100–119°	121°	114–127°	0.001
Extension deficit	27°	21–33°	18°	13–24°	0.002
ROM arc	83°	71–95°	102°	91–112°	<0.001

### Secondary outcomes

Statistically significant improvement in mean PROM-scores at three months was seen for both the OES and Quick-DASH. The OES improved from 56/100 to 65/100 (*p* = 0.01). Quick-DASH score decreased from 35/100 to 26/100 (*p* = 0.03). PSFS scores and pain scores did not significantly improve. Despite significant difference scores of the OES and the Quick-DASH, no PROM-scores surpassed the minimal clinically important difference (MCID) in our cohort (see Table 3) for an overview of all PROM-scores compared to the MCID.^29–31^

**Table 3.** Differences in PROM-scores and difference scores from questionnaires at baseline and at three months compared to MCID.

PROM type	Score range (optimal score)	Mean score at baseline (95% CI)	Mean score at 3 months (95% CI)	*p*-value	Difference scores	MCID	Patients reaching MCID (%)
OES	0–100 (100)	56 (45–66)	65 (54–77)	0.01	9.6	12.1	36
Quick-DASH	0–100 (0)	35 (24–47)	27 (15–39)	0.03	−8.7	−11.4	43
PSFS	0–10 (10)	2 (2–4)	4 (3–5)	0.3	1	1.2	50
Pain at rest	0–100 (0)	23 (11–35)	13 (3–23)	0.2	−9.9	−18	20
Pain after activity	0–100 (0)	46 (31–61)	36 (21–51)	0.2	−10.0	−18	27
Elbow grade score	0–10 (10)	5 (4–6)	6 (5–7)	0.1	1	n.d.	-

OES: Oxford Elbow Score; Quick-DASH: quick disabilities of the arm, shoulder, and hand; PSFS: patient-specific functional scale; MCID: minimal clinically important difference; n.d.: not defined in literature; PROM: patient-reported outcome measure.

**Figure 2.**
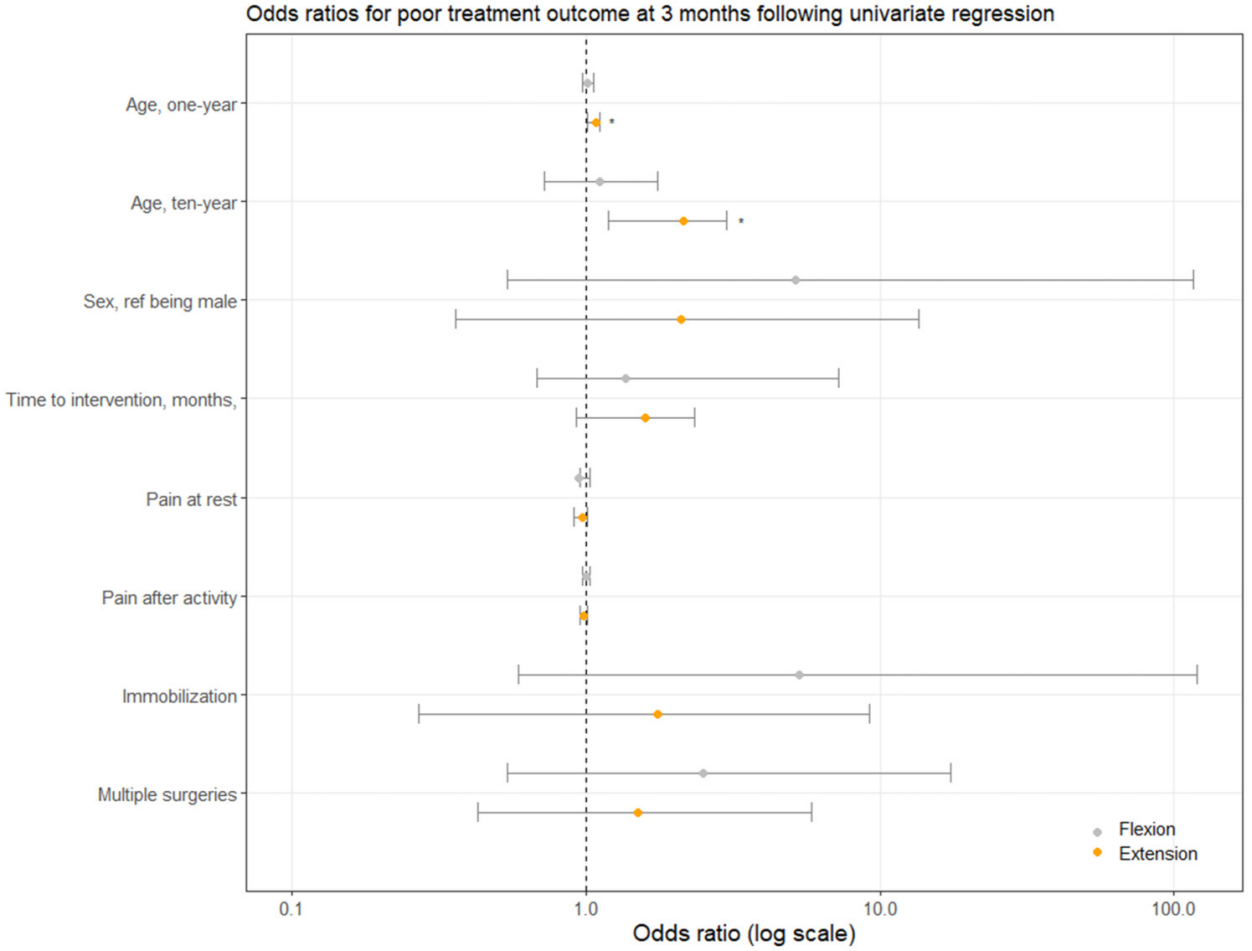
Univariate regression using generalized linear models (GLM) for poor treatment outcome using multiple predictors, listed on the y-axis. Poor treatment outcome was defined as ≤10° improvement in flexion or extension deficits at three months compared to baseline. * indicates statistically significant difference (*p* < 0.05). Grey and orange dots indicate means for flexion and extension deficits, respectively. Error bars indicate 95% confidence intervals.

In total, 56% of patients were satisfied with their treatment. This score was comprised of 22% of patients who were ‘somewhat satisfied’, 28% who were ‘very satisfied’, and 6% who were ‘utmost satisfied’. Furthermore, mean elbow grade scores improved significantly from 5/10 to 6/10 (*p* = 0.02) (Table 3). No brace-related complications were seen in either group. Besides age with an OR of 1.08 for extension deficits, no other statistically significant predictors for poor outcomes were found following univariate GLM regression analysis. The 10-year OR for age was 1.11 for flexion and 2.15 for extension deficits. Table 4 shows an overview of all values per predictor variable following univariate GLM regression analysis at three months. The values for flexion were obtained for 13 events in 23 cases, and the values for extension deficit for 14 events in 25 cases. Moreover, Figure 2 shows a graphical overview of the odds ratios per predictor, including their corresponding 95% confidence intervals.

**Table 4.** Predictor variables for poor treatment outcome at three months following univariate regression using generalized linear models (GLM).

	Flexion			Extension deficit		
	OR	95% CI	*p*-value	OR	95% CI	*p*-value
Age (per year)	1.01	0.97–1.06	0.62	1.08	1.01–1.12	0.03
Age (ten-year)	1.11	0.72–1.75	0.63	2.15	1.19–3.00	0.03
Sex (ref being male)	5.14	0.54–116	0.99	2.10	0.36–13.58	0.68
Time to intervention (months)	1.36	0.68–7.20	0.50	1.59	0.93–2.34	0.47
Pain at rest	0.95	0.96–1.03	0.76	0.96	0.91–1.01	0.12
Pain after activity	1.00	0.97–1.03	0.91	0.98	0.96–1.01	0.12
Immobilization	5.33	0.59–119	0.17	1.75	0.27–9.18	0.58
Multiple surgeries	2.50	0.54–17.46	0.22	1.50	0.43–5.87	0.80

Poor treatment outcome was defined as ≤10° improvement in flexion or extension deficits at three months.

## Discussion

In this cohort, our SPB-protocol was associated with statistically significant improvements in elbow flexion, extension deficit and total ROM-arc at three months compared to baseline. Besides improvements in elbow ROM, statistically significant improvements in mean PROM-scores were seen for both the OES and Quick-DASH questionnaires. These findings suggest that bracing for elbow contractures may prevent the need for surgical arthrolysis in some patients.

Performing an analysis at three months of bracing is relatively early, but the results are in line with previous studies with longer follow-up.^9,10,12,13^ A treatment duration of longer than three months is normally advised since it is known that the highest increase in ROM is achieved within the first six months of bracing, with further smaller increases up until one year of treatment.^13^ Despite treatment durations of SPB being up to one year in other studies, our results show that elbow ROM already significantly increases early during treatment. This implies that at three months, a decision can be made whether the patient's response to the treatment can be considered successful, and therefore, to continue brace therapy or convert to surgical arthrolysis if the patient shows <10° improvement and prevent the patient from unnecessary discomfort with brace therapy. Despite overall improvements in our cohort, a small proportion of participants retained a total ROM-arc below 100°, which is below the functional arc commonly cited for activities of daily living.^2,3^ Such patients may require longer periods of bracing or consideration of surgical arthrolysis. In the study of Lindenhovius, a protocol of three times 30 min was advised for SPB.^13^ However, for extension deficits, we believed that wearing a brace with the arm in extension was more practical during the night than during the day. Therefore, we pragmatically chose to let patients wear the turnbuckle brace during the night. Overall, 10 of the 14 patients in the post-traumatic group did not need invasive surgical treatment, and none of the patients in the post-surgical group needed subsequent surgery for residual stiffness.

Statistically significant improvements in mean PROM-scores were seen for both the OES and Quick-DASH questionnaires. However, the mean difference scores in these questionnaires did not surpass the MCID. A possible explanation for this might be pain during treatment. In a study by Lindenhovius et al., it was found that pain was an important predictor of disability and health status after elbow contracture release.^28^ In our cohort, we did not see a statistically significant decrease in pain scores, either at rest or during activities. In fact, after three months of treatment, mean pain scores after activity remained at 38/100. Moreover, patients are specifically instructed to apply stretch in the direction requiring improvement until they experience a slight discomfort. These periods of discomfort during brace wear may also contribute to the remainder of pain in periods of rest or during activity when the brace is not worn. Both the OES and the Quick-DASH have a subset of questions regarding pain, leading to the question of whether these scores might be influenced by pain during treatment or during activities. Another possible reason might simply be that the PROM-scores improve slowly over time, and therefore, three months might still be relatively short for these PROM-scores to improve sufficiently. Other studies also analyze the difference in PROM-scores at later time points.^13,28^ PROM-scores in these studies were obtained at six months and one year or at least one year after treatment. With PROM-scores improving up until one year of treatment, clinically relevant improvements might, therefore, occur in our cohort after treatment longer than three months. Despite the fact that on group-level the mean PROM-scores did not surpass the MCID, still, over one-third of the patients for the OES and almost half of the patients for the Quick-DASH did see clinically relevant improvement in their functional scores, with only three patients for the OES and two patients for the Quick-DASH performing worse. In addition, the patients in our study were included in a tertiary referral center, highly experienced in elbow pathology. This means that a significant portion of our cohort consisted of patients who had already undergone multiple prior treatment attempts. It is, therefore, even more compelling to see that these difficult-to-treat patients are able to gain both clinical and functional improvements in elbow function after only three months of treatment. In this context, the overall treatment satisfaction of 56% may actually be regarded as encouraging. Given that these patients often present with long-standing stiffness and limited expectations due to previous unsuccessful interventions, achieving satisfaction in over half of the cohort highlights the meaningful benefit that SPB can provide. These results may be even more profound when this SPB-protocol is used in the general population.

To optimize minimal invasive treatment of elbow contractures, we tried to identify patient factors that might contribute to unsatisfactory treatment outcomes of bracing and, therefore, avoid unnecessary discomfort and delay in surgical arthrolysis. We found age to be significantly associated with poor treatment outcomes in patients with extension deficits with a one-year OR of 1.08 (95% CI 1.01–1.12, *p* = 0.03). To our knowledge, this correlation has not yet been found in earlier studies. This might imply that older patients may be less responsive to brace treatment and could, therefore, possibly opt for early surgery for contracture release. The decision, however, whether to opt for surgery in older patients should also be evaluated in light of the functional goals of the patient. Older patients generally pursue a less active lifestyle and may, therefore, be satisfied with lower functional goals achievable with bracing.

Study strengths include the fact that all data was prospectively gathered and that a large part of our cohort comprises of tertiary-referred patients, which allows for testing this treatment protocol in a group that has already had multiple unsuccessful treatment attempts of treatment for their elbow stiffness. Furthermore, our institution uses a system that integrates both standard clinical follow-up time points together with the responses from routinely collected PROM questionnaires at these same time points to create a broad view of a patient's response to treatment. Study limitations include the small sample size and the absence of a control group. Due to our small sample size, it was not possible to analyze the results per treatment group. Furthermore, with our sample size, only a univariate GLM analysis of risk factors could be performed. Therefore, we could not yet test whether our association for higher age being a risk factor for poor treatment outcomes still holds after multivariate analysis. Moreover, we could not yet define which specific age groups were more susceptible for treatment-resistant stiffness as it was not possible to stratify into different age groups. In our univariate GLM analysis, there was no possibility yet of testing for interactions between risk factors or testing for confounders. We did not systematically record brace-wearing adherence, protocol fidelity or formal metrics of brace tolerability, which may have influenced both ROM and PROM outcomes. Given the absence of a control group in our study, we cannot completely isolate the treatment effects from natural recovery, regression to the mean or other confounders. It, however, does provide real-world data on treatment outcomes and add preliminary evidence in favor of SPB treatment in line with other studies.^8–12,14,15^

Future research should focus on comparing SPB with other rehabilitation modalities in a randomized controlled trial setting, exploring the long-term outcomes of SPB therapy on specific subgroups and further identifying patient-specific factors that predict response to treatment in multivariate analyses. Additionally, investigations into the optimal duration and intensity of SPB application for different types of elbow contractures could refine treatment protocols. While treatment satisfaction was measured in this study, patient satisfaction with regard to the wearing of the brace itself would be interesting to elucidate. As well as specific factors determining brace comfort, tolerability and adherence to treatment protocol. As far as the authors are aware, no study has previously recorded brace-wearing time over the course of treatment.

In conclusion, SPB is a safe treatment modality associated with early ROM improvements and functional outcomes in patients with post-traumatic or post-operative elbow stiffness. Static-progressive bracing should be considered within the standard treatment algorithm for patients with post-traumatic or post-surgical limitations in elbow flexion and/or extension, as it may prevent patients from undergoing unnecessary surgical interventions.
